# Genome-Wide Investigation of Hsf Genes in Sesame Reveals Their Segmental Duplication Expansion and Their Active Role in Drought Stress Response

**DOI:** 10.3389/fpls.2016.01522

**Published:** 2016-10-13

**Authors:** Komivi Dossa, Diaga Diouf, Ndiaga Cissé

**Affiliations:** ^1^Centre d'Etudes Régional pour l'Amélioration de l'Adaptation à la SécheresseSénégal; ^2^Laboratoire Campus de Biotechnologies Végétales, Département de Biologie Végétale, Faculté des Sciences et Techniques, Université Cheikh Anta DiopDakar, Sénégal

**Keywords:** sesame, Hsf gene family, evolution, expression profile, drought

## Abstract

Sesame is a survivor crop cultivated for ages in arid areas under high temperatures and limited water conditions. Since its entire genome has been sequenced, revealing evolution, and functional characterization of its abiotic stress genes became a hot topic. In this study, we performed a whole-genome identification and analysis of Hsf gene family in sesame. Thirty genes encoding Hsf domain were found and classified into 3 major classes A, B, and C. The class A members were the most representative one and Hsf genes were distributed in 12 of the 16 linkage groups (except the LG 8, 9, 13, and 16). Evolutionary analysis revealed that, segmental duplication events which occurred around 67 MYA, were the primary force underlying Hsf genes expansion in sesame. Comparative analysis also suggested that sesame has retained most of its Hsf genes while its relatives viz. tomato and potato underwent extensive gene losses during evolution. Continuous purifying selection has played a key role in the maintenance of Hsf genes in sesame. Expression analysis of the Hsf genes in sesame revealed their putative involvement in multiple tissue-/developmental stages. Time-course expression profiling of Hsf genes in response to drought stress showed that 90% Hsfs are drought responsive. We infer that classes B-Hsfs might be the primary regulators of drought response in sesame by cooperating with some class A genes. This is the first insight into this gene family and the results provide some gene resources for future gene cloning and functional studies toward the improvement in stress tolerance of sesame.

## Introduction

To cope with environmental stresses, plants have developed complex transcriptional systems that involve transcription factors (Mittler, 2006). Abiotic stresses in plants induce physiological and biochemical changes controlled by regulation of gene expression. Transcription Factors (TFs) are important for regulating gene expression in responses to biotic or abiotic stimuli. Among these TFs, WRKY (Rushton et al., 2010), MYB (Dubos et al., 2010), AP2/ERF (Mizoi et al., 2012), NAC (Puranik et al., 2012), bZip (Xu et al., 2012) and heat shock transcription factors (Hsfs) (Mittal et al., 2009) participate in these complex and overlapping processes (Wang J. et al., 2014). According to Rabara et al. (2014), TFs are strong candidates for the development of next generation of transgenic crops.

Hsfs constitute an important gene family involved in plant growth and development, as well as in responses to abiotic stresses (von Koskull-Döring et al., 2007). Studies revealed that a typical Hsf consists of five conserved motifs, including a DNA-binding domain (DBD) connected to an oligomerization domain (OD) consisting of hydrophobic heptad repeats (HR-A and HR-B). Beside DBD and OD, a nuclear localization signal (NLS), a nuclear export signal (NES) and an activator peptide motif (AHA) (Mittal et al., 2009; Scharf et al., 2012) are also present. Based on the structural characteristics of their HR-A/B domain and phylogenetic comparisons, plant Hsf genes may be divided into 3 classes: “A”, “B”, and “C” (Nover et al., 2001; Baniwal et al., 2004). The Class “A” and “C” Hsfs contain an extended HR-A/B with 21 and 7 amino acid residues between the HR-A and HR-B region, respectively. In contrast, class B Hsf genes lack an insertion in the HR-A/B region (Nover et al., 2001; Baniwal et al., 2004). Additionally, the class “A” Hsf genes contain AHA activation domains that are absent in class “B” and “C” Hsf genes (Döring et al., 2000). Under non-stress condition, Hsf genes are maintained in inactive state and form cytoplasmic complexes with Hsp90/Hsp70 chaperone complexes (Lin et al., 2011). When activated, Hsf genes are released from chaperone complexes, recognize the conserved binding motifs (heat shock elements, HSEs) within the promoters of Hsf-responsive genes (Bienz and Pelham, 1987) and undergo phosphorylation, sumoylation, trimerisation and nuclear import (Baniwal et al., 2004; Scharf et al., 2012). Class A Hsfs are involved in transcriptional activation and environmental stress responses (Shim et al., 2009), while Hsfs in class B lack the AHA activator domain and serve as repressors of gene expression (Ikeda et al., 2011) or trancriptional coactivators with class A Hsfs (Wang J. et al., 2014). So far, there is no information regarding the functions of class C members.

Due to the importance of this gene family, it has been extensively studied in many crops such as *Arabidopsis* (Nover et al., 2001), rice (Guo et al., 2008; Chauhan et al., 2011), maize (Lin et al., 2011), apple (Giorno et al., 2012), legume (Lin et al., 2014), cabbage (Song et al., 2014), cotton (Wang J. et al., 2014), Chinese white pear (Qiao et al., 2015), Chinese pepper (Guo et al., 2015), strawberry (Hu Y. et al., 2015) etc. Even though Hsf genes were first described as heat stress response genes, many studies have so far reported their implication in the responses to other abiotic stresses such as drought (Miller and Mittler, 2006; Swindell et al., 2007; Hu et al., 2009; Chauhan et al., 2011). Wang et al. (2007) reported that Hsf8-like gene was activated in response to dehydration in rice cultivars. Furthermore, 12 Hsf genes were up-expressed in rice under drought stress (Moumeni et al., 2011). Later on, Bechtold et al. (2013) demonstrated that the over-expression of the Hsf class A1B genes triggers drought resistance in *Arabidopsis*. Hwang et al. (2014) demonstrated that HsfA6a is involved in drought stress response in *Arabidopsis*. More recently, Reddy et al. (2014) confirmed the role of the Hsf gene “HvHsfB2c” in drought stress response in Barley. Hence, Hsf gene family stands as a good candidate to investigate the drought tolerance in crops.

Sesame (*Sesamum indicum* L.) is, surprisingly, one of the most drought-tolerant species within the major crops of *Asteridae* subclass (Dossa et al., 2016a). It is mainly grown in arid and semi-arid areas with high temperatures and frequent occurrence of drought (Eskandari et al., 2009). Even though sesame naturally exhibits relatively good tolerance to drought, its productivity is highly weakened by water scarcity (Bahrami et al., 2012). Drought is one of the most important constraints in sesame production (Betram et al., 2003; Pathak et al., 2014). Sun et al. (2010) demonstrated that sesame is highly sensitive to drought during flowering stage and its yield is adversely affected as the level of drought increases (Hassanzadeh et al., 2009). In addition, some reports revealed the negative effect of drought on both the quality and quantity of sesame oil (Kim et al., 2006; Ozkan and Kulak, 2013; Kadkhodaie et al., 2014).

Sesame seed is highly valued because it is a source of excellent vegetable oil and has one of the highest oil contents (~55%) among major oil crops (Pathak et al., 2014). Its oil contains sesamin, sesamolin, and sesamol antioxidants; hence, it is very stable and has a long shelf life (Were et al., 2006). Due to the increasing demand for vegetable oils which is expected to reach 240 million tons by 2050 (Barcelos et al., 2015), breeding the sesame varieties with greater drought tolerance is drawing great attention from breeders (Dossa et al., 2016a). Hence, functional characterization of Hsf genes under drought stress could help to identify some candidate genes that improve drought tolerances in sesame. Recent release of the full genome sequence of sesame (Wang et al., 2015) has provided the useful genomic platform for genome-wide survey of this gene family.

Here, we report an extensive picture of the Hsf gene family in sesame. First, we identified and characterized Hsf genes in the sesame genome. Secondly, we performed phylogenetic and evolutionary analyses. Finally, the expression profiling of Hsfs in different organs and under drought stress were assessed.

## Materials and methods

### Identification and classification of Hsfs in sesame genome

The Hsf genes sequences of *Arabidopsis* and *Utricularia gibba* were retrieved from the Plant Transcription Factor DataBase (http://planttfdb.cbi.pku.edu.cn/) (Jin et al., 2014). Based on the genome and proteome sequences of sesame downloaded from the Sinbase (http://ocri-genomics.org/Sinbase/) (Wang et al., 2014), an extensive search was performed to identify all members of the Hsf gene family. Using HMMER3 tool of the software Unipro UGENE (Okonechnikov et al., 2012), the Hsf domain, denominated as PF00447 in the Pfam HMM library (http://Pfam.sanger.ac.uk/) (Finn et al., 2014), was searched against the sesame proteome.

The SMART program (Letunic et al., 2015) and Pfam were used to confirm the signature DBD domain of Hsfs (Bateman et al., 2004). All candidate Hsf genes with no DBD were removed. Additionally, the MARCOIL program (Delorenzi and Speed, 2002) was used to detect the coiled-coil structures and eliminate any sequence without a coiled-coil structure. The physical and chemical parameters of the retained Hsf genes were analyzed using ProtParam tool (http://web.expasy.org/protparam/). The classification of candidate Hsf proteins into three different classes A, B, and C was performed by Heatster (http://www.cibiv.at/services/hsf/) (Scharf et al., 2012) based on the information of oligomerization domains (Nover et al., 2001).

### Phylogenetic analysis, structure, and motif identification of Hsfs

To understand the evolutionary relationships between Hsf genes in sesame, *Arabidopsis* and *U. gibba*, multiple alignments of the N-proximal regions of Hsf proteins from the three species were conducted using ClustalW program built in the MEGA 6.0 software (Tamura et al., 2013) with a gap open penalty of 10 and gap extension penalty of 0.2. The result of alignment was used for the construction of an un-rooted Maximum-Likelihood (ML) tree with a 1000 bootstrap value.

Based on Sinbase information, exon-intron substructure map was constructed by the Gene Structure Display Server (GSDS 2.0), web-based bioinformatics tool (http://gsds.cbi.pku.edu.cn/) (Hu B. et al., 2015). Conserved motifs of Hsf proteins were analyzed using MEME Suite version 4.10.2 (Bailey et al., 2009) with the parameters set as follows: minimum width of 6, maximum width of 50, the maximum number of motifs was set to 20 and the number of repetitions set to “any number”. All other parameters were set at default.

### Chromosomal location, gene duplication and comparative mapping of orthologous Hsf genes in sesame and *Arabidopsis*

The Hsf genes were mapped onto the 16 Linkage Groups (LGs) of sesame genome on the basis of the information from Sinbase using the software MapChart 2.3 (Voorrips, 2002). The duplicated genes were identified and connected by lines according to Lin et al. (2011). As regard to estimating the synonymous (Ks) and non-synonymous (Ka) substitution rates, the cDNA and the protein sequences of the duplicated genes pairs were submitted to PAL2NAL (http://www.bork.embl.de/pal2nal/) (Suyama et al., 2006). The approximate date of the duplication event was calculated as described by Wang et al. (2015).

In addition, the amino acid sequences of predicted Hsf proteins were BLASTp searched against protein sequences of *Arabidopsis* in NCBI. Hits with *E*-value ≥ 1e-40 and at least 70% similarity were considered significant. The relationships between the orthologous genes of sesame and *Arabidopsis* were plotted using Circos (http://circos.ca/) (Krzywinski et al., 2009).

### Identification of SSR markers and interaction network of Hsfs

The presence of simple sequence repeats (SSRs) were searched against the DNA sequences of the Hsf genes using the web based software Websat (http://wsmartins.net/websat/) (Martins et al., 2009) with the following parameters: two to six nucleotide motifs were considered and the minimum repeat unit was defined as five reiterations for di-nucleotides and four reiterations for other repeat units (Dossa et al., 2016b). Furthermore, specific protein interaction networks based on *Arabidopsis* interactome were constructed applying STRING software (http://string-db.org/) (Szklarczyk et al., 2011).

### Expression analysis of Hsf genes in different organs using RNA-seq data

To analyze the expression patterns of sesame Hsf genes, we used RNA-seq data from root, stem, seeds at different stages and leaf downloaded from SesameFG (http://202.127.18.220/hg/). Reads per kilobase per million mapped reads (RPKM) values were log10 transformed and, finally, the heat map of hierarchical clustering was constructed using the MEV Software (Saeed et al., 2006).

### qRT-PCR analyses of Hsf genes expression profiles under drought stress

#### Plant materials and stress treatment

Seeds of 02 contrasting sesame accessions (LC164-drought tolerant) and (hb168-drought sensitive) previously studied by Boureima et al. (2012) were sown in pots (25 cm diameter and 30 cm depth) filled with a mixture of soil, sand, and compost (5:2:2, v/v/v). The seeds were mixed with calthio C fungicide (2 kg/ha) prior to sowing in an in-walk growth chamber. The seedlings were thinned at 2 true leaves stage and one plant was kept per pot. They were watered daily with 75 ml of tap water during 30 days before being exposed to drought stress. Water was withheld for 6 days and re-supplied again from the 7th day. The mean temperature during the experiment was about 33°C. To assess the Hsf gene expression, root samples from single plant were collected under drought stress at day 0, 3, 6, and after re-watering at day 9 corresponding to 35% soil volumetric water content (vwc), 16, 9, and 35% vwc, respectively. Three biological samples were immediately after harvesting frozen in liquid nitrogen and stored at −80°C prior to RNA extraction.

#### RNA extraction and qRT-PCR analysis

RNA extraction was performed according to the procedure described by Wei et al. (2011). RNAprep Pure Plant Kit (Tiangen) was used to isolate total RNA from each frozen sample and its concentration and quality were tested using an ultraviolet spectrophotometer and denaturing agarose gels (1%). One microgram of RNA was reverse transcribed using the Superscript III reverse transcription kit (Invitrogen) according to the manufacturer's instructions. Gene-specific primers were designed by using Primer5.0 (Lalitha, 2000; Supplementary Table 1). qRT-PCR was performed in triplicate and sesame housekeeping gene actin7 (*SIN_1006268*) (Dossa et al., 2016b) was used as a reference for normalization following the 2^−ΔΔCt^ method (Livak and Schmittgen, 2001). Data were presented as means ± SD and analyzed using one-way analysis of variance (ANOVA) with Minitab® 16 Software. The partitioning of the means was made with Tukey test at 5% probability level.

## Results

### Identification, physical location and duplication analysis Hsfs in sesame genome

A total of 30 Hsf genes were confirmed in sesame genome with apparently complete Hsf-type DNA-binding domains and oligomerization domains ranging from 255 to 2646 bp in length (Table 1, Supplementary Table 2). The deduced proteins sizes vary from 84 amino acids (*HSF9*) to 889 amino acids (*HSF21*) and the isoelectric point ranging from 4.73 (*HSF6*) to 9.45 (*HSF25*). Table 2 provides more detailed information about each protein physicochemical characteristics.

**Table 1:** **Summary of Hsf genes identified in the sesame genome**.

**Gene name**	**Gene locus ID**	**Linkage group**	**ORF length (bp)**	**CDS**	**Group**	**Start site (bp)**	**End site (bp)**
HSFsi1	SIN_1013653	LG1	1047	2	C1	10142893	10145091
HSFsi2	SIN_1017828	LG1	966	2	B2	6756792	6758284
HSFsi3	SIN_1021512	LG1	1263	3	A2	1999558	2001510
HSFsi4	SIN_1018328	LG2	993	2	C1	16210245	16212448
HSFsi5	SIN_1021084	LG2	1305	1	A1	12737745	12739049
HSFsi6	SIN_1021280	LG2	1344	2	A8	14639472	14641759
HSFsi7	SIN_1011428	LG3	882	3	B4	22028397	22029694
HSFsi8	SIN_1015377	LG3	1248	2	A5	17632845	17634433
HSFsi9	SIN_1015378	LG3	255	1	A5	1763831	17638564
HSFsi10	SIN_1021859	LG3	1032	2	A6	14669967	14673393
HSFsi11	SIN_1016568	LG4	1041	2	B2	10103399	10104533
HSFsi12	SIN_1018625	LG4	738	2	B3	2626922	2628871
HSFsi13	SIN_1023515	LG5	708	2	B3	1314659	13148692
HSFsi14	SIN_1012376	LG6	1083	2	A7	1879296	1880955
HSFsi15	SIN_1012869	LG6	1107	2	A7	21093626	21095081
HSFsi16	SIN_1015574	LG6	936	2	B2	8664075	8665158
HSFsi17	SIN_1022384	LG6	1323	2	A1	23239742	23241640
HSFsi18	SIN_1009110	LG7	909	2	B2	8529389	8530389
HSFsi19	SIN_1009271	LG7	1425	2	A1	9553488	9555700
HSFsi20	SIN_1011726	LG7	1044	2	B4	4967228	4968418
HSFsi21	SIN_1009981	LG10	1149	2	A6	102675	104434
HSFsi22	SIN_1012975	LG11	1380	2	A3	11985049	11987041
HSFsi23	SIN_1024732	LG11	1314	3	A2	8776734	8778561
HSFsi24	SIN_1001354	LG12	2646	12	B2	3805133	3811746
HSFsi25	SIN_1005270	LG12	603	2	B1	4222007	4222997
HSFsi26	SIN_1019308	LG14	1212	2	A1	2693425	2695051
HSFsi27	SIN_1004844	LG15	816	2	B1	2925788	2928984
HSFsi28	SIN_1001533	Scaffold00164	1044	2	B2	241403	242576
HSFsi29	SIN_1001536	Scaffold00164	1281	2	A4	250746	252144
HSFsi30	SIN_1001483	Scaffold00165	1617	2	A3	213387	215706

**Table 2:** **Physicochemical characteristics of sesame Hsf genes**.

**Gene name**	**Size (aa)**	MW ^*^ (KDa)	PI^*^	AI^*^	GRAVY^*^
HSFsi1	348	38.9395	5.31	62.24	−0.628
HSFsi2	321	34.6765	5.11	69.66	−0.559
HSFsi3	420	47.0955	4.88	75.64	−0.553
HSFsi4	330	37.6515	6.03	70.58	−0.593
HSFsi5	434	47.2389	4.82	71.91	−0.561
HSFsi6	447	50.7831	4.73	71.5	−0.681
HSFsi7	293	32.7347	6.83	68.87	−0.601
HSFsi8	415	46.6999	5.74	72.31	−0.791
HSFsi9	84	9.1413	7.88	69.64	−0.046
HSFsi10	343	39.1296	5.61	67.87	−0.74
HSFsi11	346	38.0552	5.54	65.43	−0.672
HSFsi12	245	28.1158	6.62	70.82	−0.747
HSFsi13	235	26.3185	6.15	71.36	−0.654
HSFsi14	360	41.6498	5.58	66.31	−0.839
HSFsi15	368	42.708	5.51	67.55	−0.866
HSFsi16	311	34.2703	6.08	66.62	−0.655
HSFsi17	440	50.2907	5.13	72.64	−0.76
HSFsi18	302	33.821	5.53	80.5	−0.522
HSFsi19	474	52.3134	5.44	70.49	−0.569
HSFsi20	347	38.8428	8.06	73.92	−0.5
HSFsi21	382	43.4697	5.24	68.38	−0.752
HSFsi22	459	50.9762	5.12	67.34	−0.498
HSFsi23	437	49.8496	5.67	81.01	−0.541
HSFsi24	881	99.2237	5.83	81.09	−0.377
HSFsi25	200	23.4331	9.45	63.95	−0.692
HSFsi26	403	45.6087	5.21	72.8	−0.674
HSFsi27	271	30.0876	6.96	60.44	−0.758
HSFsi28	347	40.0627	4.93	63.8	−0.843
HSFsi29	426	48.0975	5.09	71.62	−0.723
HSFsi30	538	59.4942	5.05	61.49	−0.625

* PI: Isoelectric point, MW: Molecular Weight, AI: Aliphatic Index, GRAVY: Grand Average of Hydropathy.

**Figure 1:**
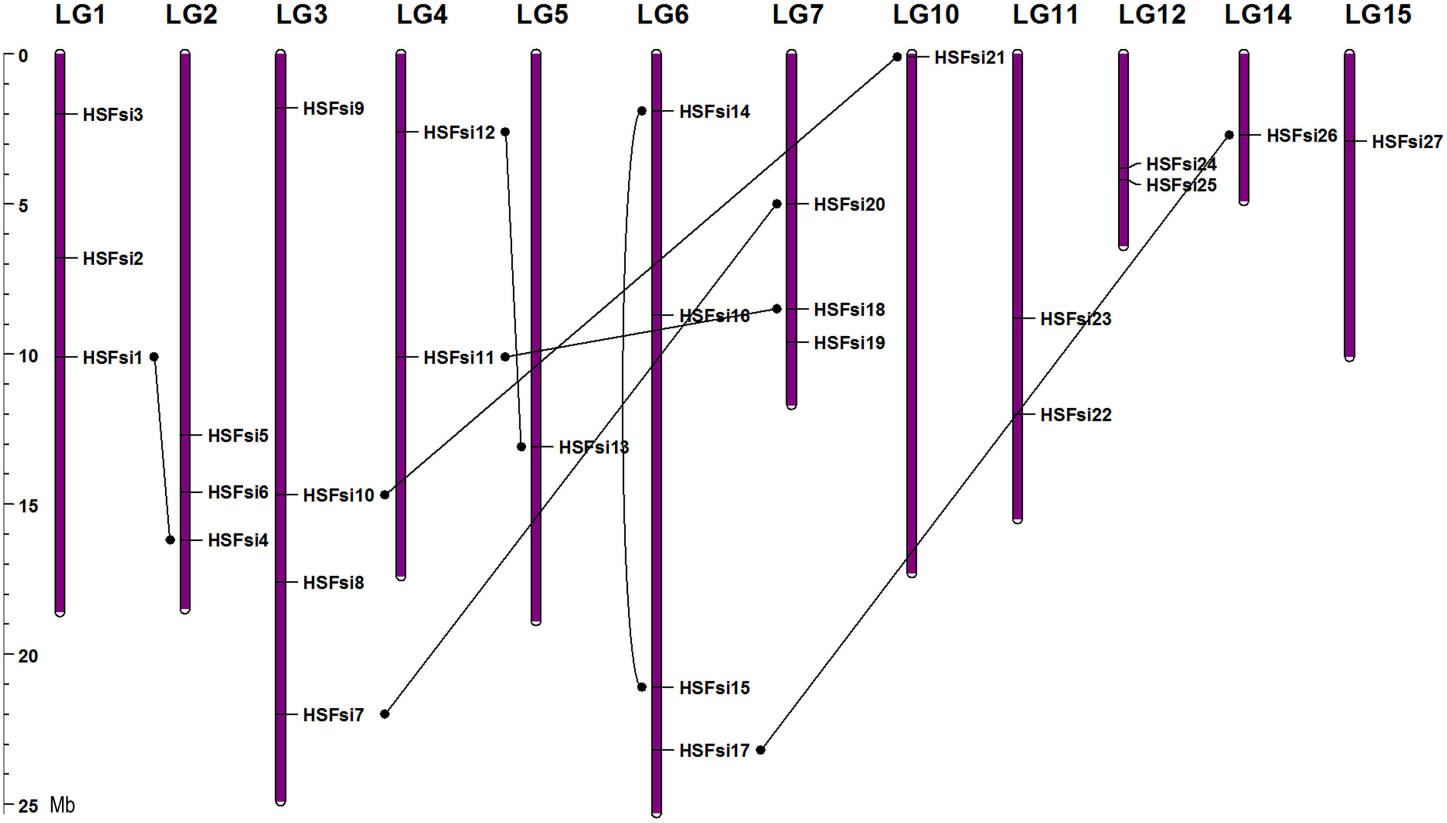
**Mapping of sesame Hsf genes based on their physical positions**. Vertical bars represent linkage groups (LG) of the sesame genome. The LG numbers are indicated at the top of each LG. Black lines linked duplicated genes.

Except for 3 genes (*HSF28, HSF29*, and *HSF30*) which were located on the unanchored scaffolds that could not be mapped to a specific LG, all the Hsf genes were unevenly distributed among 12 LGs out of the 16 LGs of sesame genome, suggesting that Hsf genes may have been widely distributed in the genome of the common ancestor (Figure 1). The numbers of sesame Hsf genes in each LG vary widely with LG3 and LG6, harboring the highest number of Hsf genes (3; 10%) while LG5, LG10, LG14, and LG15 have only one gene (3.33%).

Analyzing of the duplication events can help to elucidate the evolution mechanisms of the sesame Hsf gene family. In total, 7 duplicated gene pairs across 11 LGs were identified including six segmental duplication events between different LGs (*HSF1* and *HSF4, HSF12* and *HSF13, HSF11* and *HSF18, HSF10* and *HSF21, HSF7* and *HSF20, HSF17* and *HSF26*) and one duplication event within the same LG6 (*HSF14* and *HSF15*). The duplicated gene pairs are linked with black lines in Figure 1.

**Figure 2:**
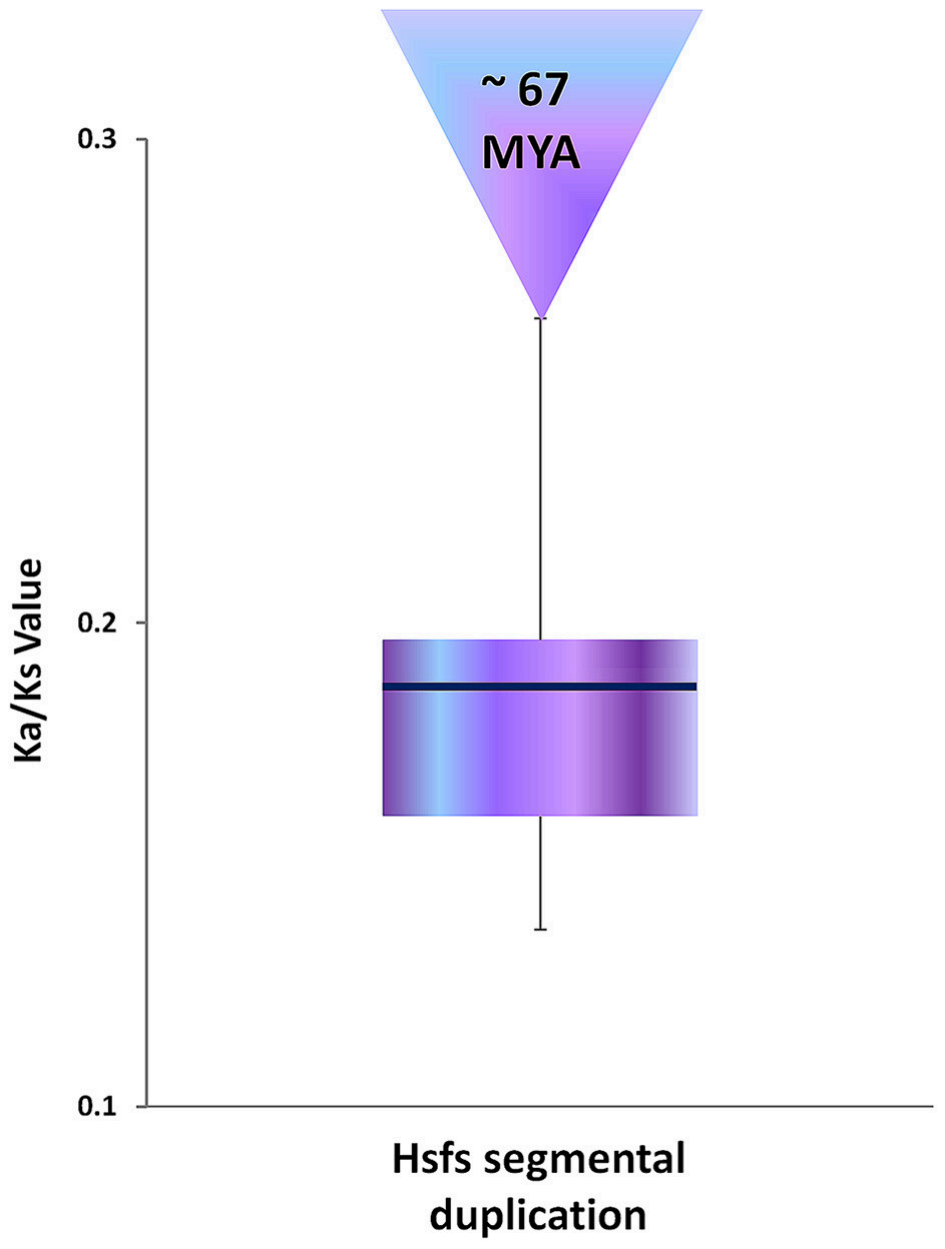
**Distribution of synonymous substitution rate (Ks) and estimated of time of duplication (MYA) based on sesame Hsf duplicated genes**.

To get more insights into the evolution of these duplicated genes, synonymous (Ks), non-synonymous (Ka) and their ratio (Ka/Ks) values were used to examine the selective pressure and duplication time of the 7 sesame Hsf segmentally duplicated genes. In general, a Ka/Ks > 1 means positive selection, Ka/Ks < 1 indicates purifying selection, and Ka/Ks = 1 stands for neutral selection (Nekrutenko et al., 2002). The Ka/Ks ratio for all sesame Hsf duplicated genes ranged from 0.1367 to 0.263, thus Ka/Ks < 1 for all the 7 duplicated gene pairs (Supplementary Table 3). These results suggested that the duplicated Hsf genes were under strong purifying selection pressure. In addition, the duplicated events were estimated to have occurred approximately ~67 million year ago (51.5–93.7 MYA; Figure 2).

### Conserved domains and genes structure of Hsfs in sesame

All the 30 Hsf genes were submitted to Heaster program to uncover the conserved functional domains. The details of various functional domains such as DBD, HR-A/B, NLS, NES, and AHA motifs are presented in Table 3. As the most conserved domain in the Hsf genes, DBD composed of approximately 90 amino acids was found in all the 30 genes. In addition to DBD, HR-A/B, another core conserved domain, was also present in all sesame Hsf proteins, while the other conserved domains were specific to each class. According to the distinction between the HR-A and HR-B motifs, Hsfs were divided into A, B, and C classes. The class A was the most representative one with 16 genes out of 30 genes. Twelve genes belonged to the class B and the remaining 2 sesame Hsf genes were classified into the class C.

**Table 3:** **Functional domains of sesame Hsf genes**.

**Gene Name**	**Group**	**DBD**	**HR-A/B**	**NLS**	**NES**	**AHA1**	**AHA2**
HSFsi17	A1	11–104	116–165	(201) KKRR	(427) LTQQIGHL	(249) LTFWEKFVHG	(379) DVFWQQFLTE
HSFsi19	A1	19–112	134–198	(223) KKRR	(460) LTQQMGLL		(411) DVFWEQFLSG
HSFsi5	A1	1–38	62–126	(152) KKRR	(420) KLTEQMELL		(372) DPFWEKFLQS
HSFsi26	A1	11–104	116–175	(201) KKRR	(378) DVFWQQFLTE	(249) LTFWENFMHV	
HSFsi3	A2	43–136	152–216	(294) HKRR		(319) TLFSAALDD	(359) SEW
HSFsi23	A2	72–190	214–264	(275) LFLQHCL	(405) IVSELENLI	(379) FVLWEKLMED	
HSFsi22	A3	63–161	190–236	(262) RSMRKFVKHQ		(295) EDVWNTG	(414) KFWSNL
HSFsi30	A3	111–204	233–279	(305) RATRKFVKHQ		(457) EDSFWGT	(476) TELWST
HSFsi29	A4	11–104	128–185	(203) RKRR	(413) LAEQLGHL	(255) LTFWEKIAFE	(362) DVFWEQFLTE
HSFsi8	A5	61–118	129–144			(364) DVFWEQFLTER	
HSFsi9	A5	17–76	101–169	(188) KR			
HSFsi10	A6	40–133	149–213	(234) KRRKK	(328) VDVLAEQMGFL	(303) KGLWEELLND	
HSFsi21	A6	38–131	147–211	(236) KKRRK	(367) VDAFTDQLGFL		(343) EGFWGGIEDE
HSFsi14	A7	48–141	157–221	(246) KKRRR	(345) VRVLADRLGFL	(317) QGFWEELLNE	
HSFsi15	A7	51–144	161–225	(250) KKRRR	(350) VNVLADRFGFL	(321) QGFWEELLNE	
HSFsi6	A8	12–119	149–206	(364) PTGTQQLVE		(350) EDDTMLEQLL	
HSFsi25	B1	9–110	149–189				
HSFsi27	B1	7–100	154–191	(230) KKKRG			
HSFsi2	B2	24–117	191–227	(279) KRLKK			
HSFsi11	B2	24–117	176–212	(290) KRGRE			
HSFsi16	B2	25–118	184–220	(269) SKRLK			
HSFsi18	B2	10–103	158–194	(260) KRGRE			
HSFsi24	B2	640–732	756–806	(822) FI	(842) AFNSLVDLI		
HSFsi28	B2	40–132	156–206	(222) KD	(321) IEIQREFLI		
HSFsi12	B3	24–117	156–191	(228) KR			
HSFsi13	B3	24–117	160–195				
HSFsi7	B4	22–115	151–212	(259) KRLH	(276) LVLEKDDLGLNLM		
HSFsi20	B4	22–115	172–208	(312) KRLH	(330) LVLEKDDLGLNLM		
HSFsi1	C1	14–108	127–170	(195) EKKRRI			
HSFsi4	C1	11–105	125–168				

**Figure 3:**
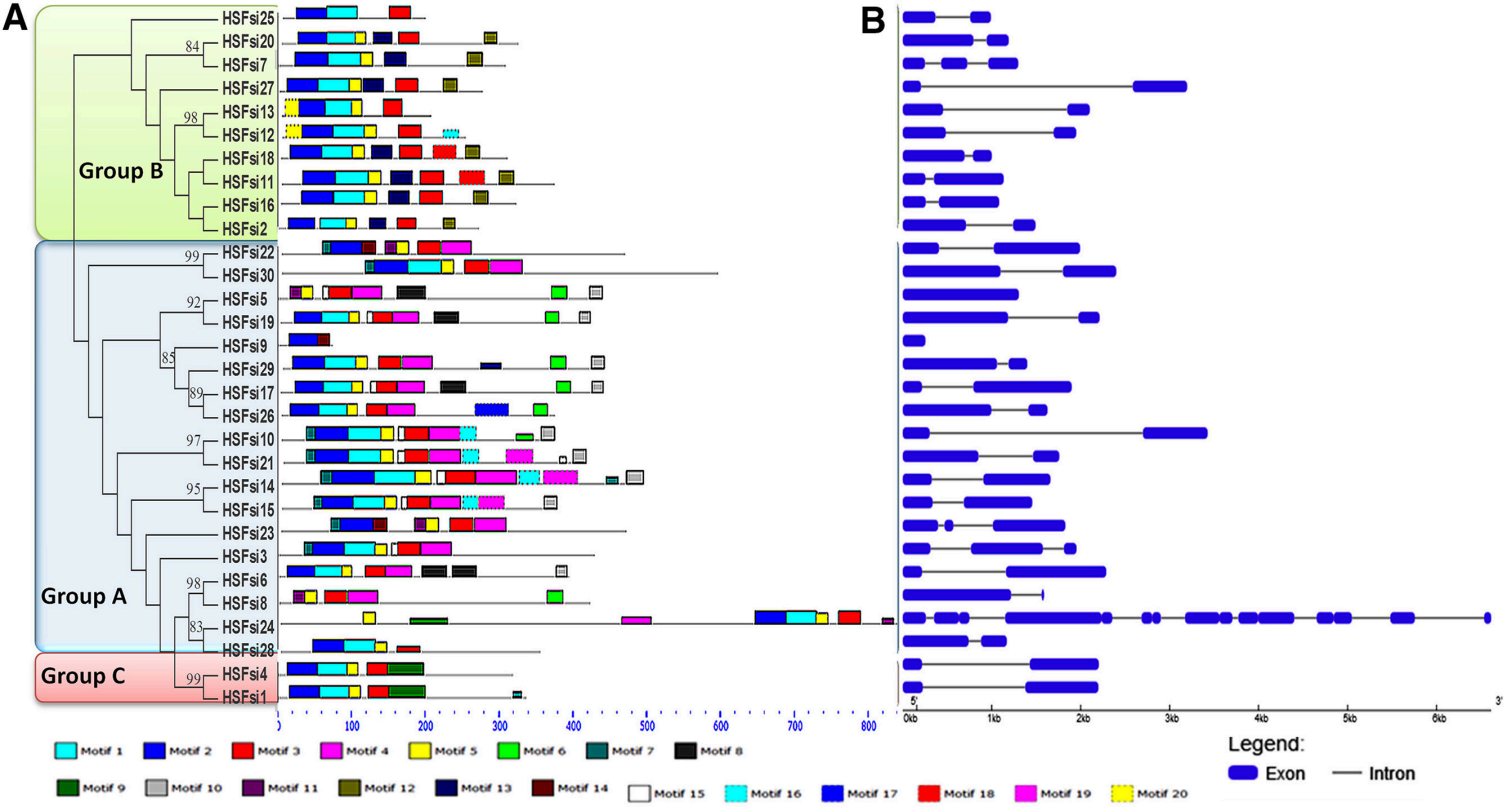
**(A)** Motifs identified by MEME tools in sesame Hsf protein sequences according to each class. Twenty motifs were identified and indicated by different colors. Motif location was showed. **(B)** Gene structures of Hsf proteins according to each class. Exons and introns are represented by blue boxes and black lines, respectively.

MEME web server was used to verify the results of domain prediction (Figure 3A; Supplementary Table 4). Twenty different motifs were found distributed throughout the Hsf proteins sequences, lengths ranging from 8 to 50 amino acids. Most of sesame Hsf genes displayed motifs 1, 2, and 5 which corresponded to the DBD domain whereas some motifs were characteristic of each class. Moreover, each class exhibited different motif organizations and two closely clustered genes on the tree generally displayed similar motif patterns. Overall, despite variability in size and sequence, the predicted Hsf DBD, HR-A/B region and other conserved domains were cross-confirmed in each sesame Hsf gene by the two combined methods.

Analysis of the exon/intron boundaries of sesame Hsf genes coupled with a phylogenetic tree exhibited a highly conserved exon/intron organization in 24 genes out of the 30 Hsf genes possessing strictly two exons. In addition, we identified 2 intronless genes (*HSF9* and *HSF5*), 3 genes possessing 3 exons (*HSF3, HSF7*, and *HSF23*) and the intriguing gene *HSF24* displaying up to 12 exons (Figure 3B). *HSF24* might contain the original genes (Nuruzzaman et al., 2010). In general, although most closely clustered genes in the phylogenetic tree displayed similar numbers of exon, there is a wide variation among the organizations and lengths of exon/intron.

### Phylogenetic analysis and mapping of orthologous genes

**Figure 4:**
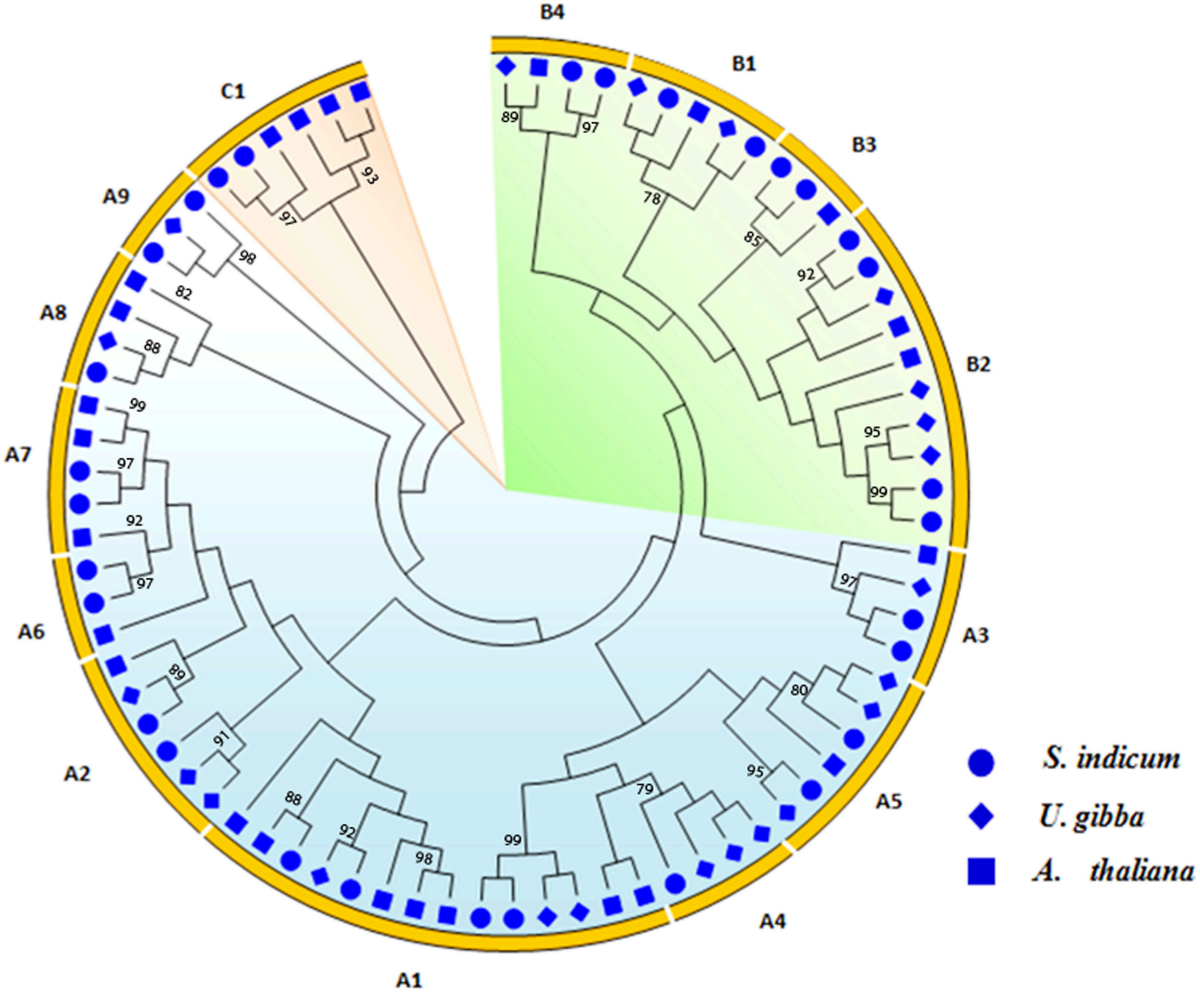
**Maximum likelihood tree of Hsf proteins in sesame, ***Arabidopsis*** and ***Utricularia gibba*****. Bootstrap values ≥75% are shown.

To evaluate the evolutionary relationships of sesame Hsf genes, phylogenetic analysis was performed based on the full-length amino acid sequences of Hsf proteins from sesame, *U. gibba* and *Arabidopsis*. The tree clearly distinguished the three classes, namely A, B, and C (Figure 4). Based on the classification of the Hsf genes of the well-studied crop *Arabidopsis*, the different functional groups belonging to each class have been identified. The class A members were further divided into 8 subclasses (A1–A8) with the subclass A1 more represented (4 members). The class B members were also classified into 4 subclasses namely B1, B2, B3, and B4, while all of the class C members were found to belong to the same subclass C1.

**Figure 5:**
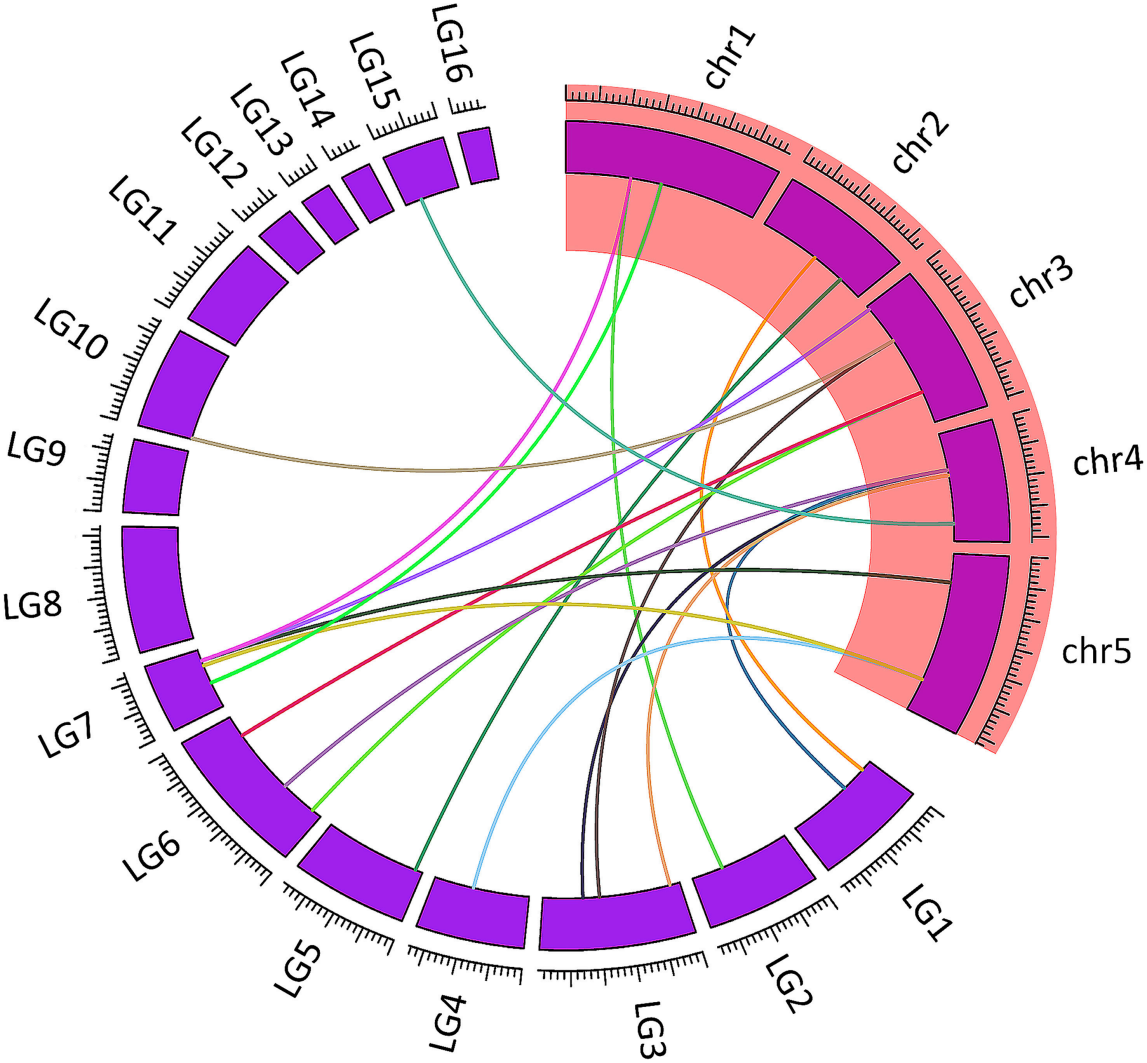
**Orthologous relationships of Hsf genes between sesame and ***Arabidopsis*** genomes**. Violet bars represent the LG of sesame genome and the chromosomes (chr) of *Arabidopsis*. The chromosomes of *Arabidopsis* are highlighted in orange. The colorful lines linked orthologous genes.

Synteny analysis of sesame Hsf genes compared to those of *Arabidopsis* could provide more functional insight. Hence, we further performed a genome-wide comparative analysis to identify orthologous Hsf genes between sesame and *Arabidopsis* genomes. In total, 18 orthologous gene pairs were found across 9 LGs in sesame (Figure 5). The LG3, LG6, and LG7 exhibited the largest orthology (3 gene pairs) while the least orthologous genes (1 gene pair) were detected on LG2, LG4, LG5, LG10, and LG15. The orthologous gene pairs and their localization in each genome are presented in Supplementary Table 5. Out of the three classes of sesame Hsf genes, only two classes (A and B) were represented in the orthologous gene pairs. Moreover, 5 Hsf genes in *Arabidopsis* genome retained 2 genes in the sesame genome while conversely, only one gene in sesame genome (*HSF19*) conserved a tripled copy in *Arabidopsis* genome (*AT5G16820, AT3G02990*, and *AT1G32330*).

### Interaction network of sesame Hsf proteins and mining of SSR markers

**Figure 6:**
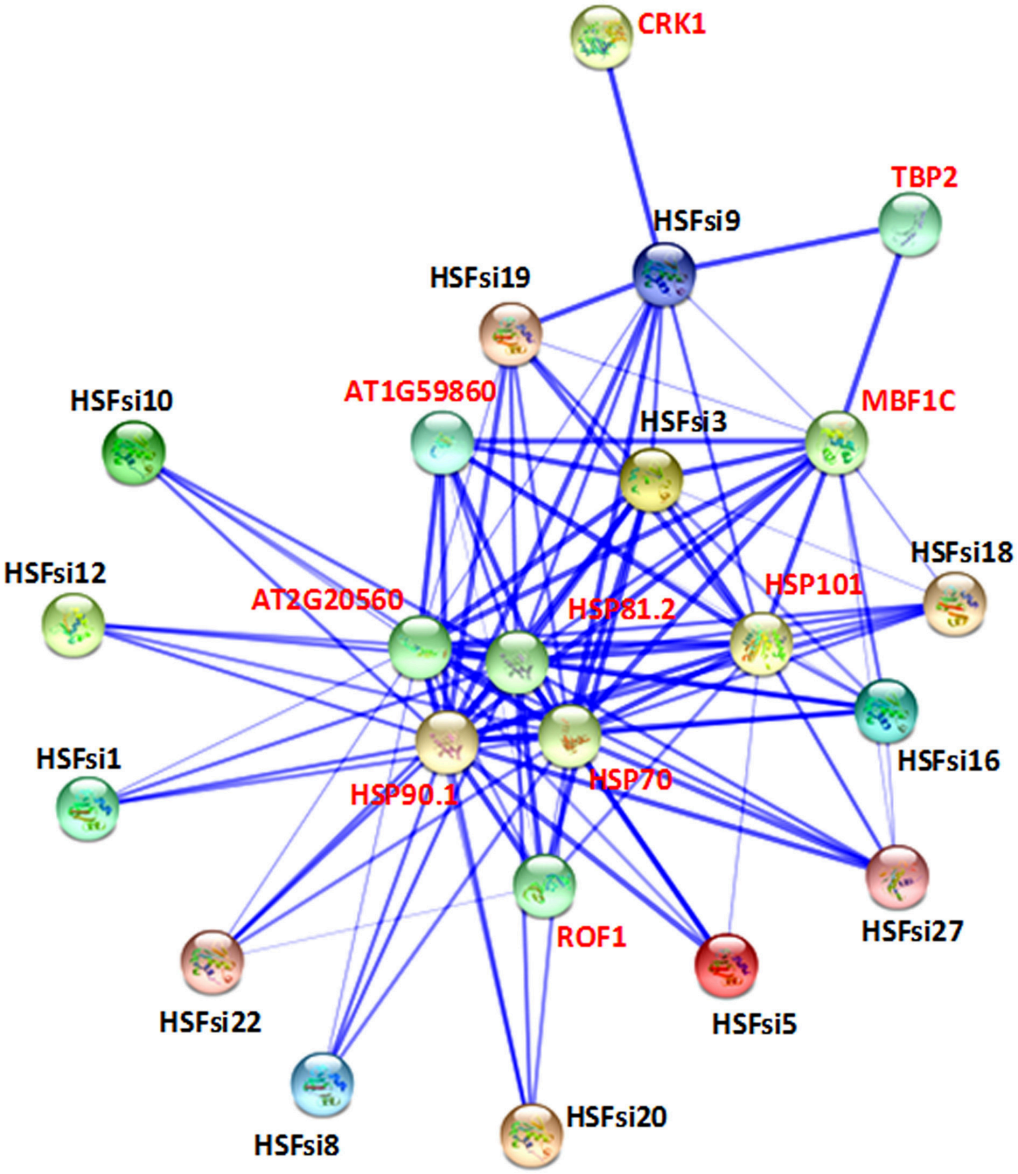
**Interaction network of 13 Hsf genes identified in sesame and related genes in ***Arabidopsis*****.

The protein–protein interaction network is a helpful preface to explore the biological functions of unknown proteins. Based on the interactome of *Arabidopsis*, the protein–protein interactions, including functional and physical interactions among Hsf genes of sesame were predicated (Figure 6). Thirteen sesame Hsf genes were involved in different interaction patterns. The *Arabidopsis* genes found in this network are mostly described to be related to defense, plant development, proteins folding and abiotic stress related genes. Hence, we hypothesized that the sesame Hsf genes might also be involved in similar function pathways. The first group including 11 sesame Hsfs (*HSF1, HSF10, HSF12, HSF22, HSF8, HSF20, HSF5, HSF27, HSF16, HSF3*, and *HSF18*) interact directly with HSP genes, hence they might be some positive or negative regulators of HSP genes in responses to abiotic stresses and plant defenses. The 2 genes *HSF19* and *HSF9* seemed to be involved in the transcription regulation of other Hsf genes.

Based on the importance of Hsf genes in responses to various environmental stresses, tagging these useful genes could help in improving marker-aided breeding and establishing better crop-improvement strategies. Out of the 30 genes, only 7 SSR markers covering 4 LGs were identified (Supplementary Table 6). Two SSR types were observed: di-nucleotide and the most abundant one, tri-nucleotide motifs.

### Organ-specific expression profiling of Hsf genes in sesame

**Figure 7:**
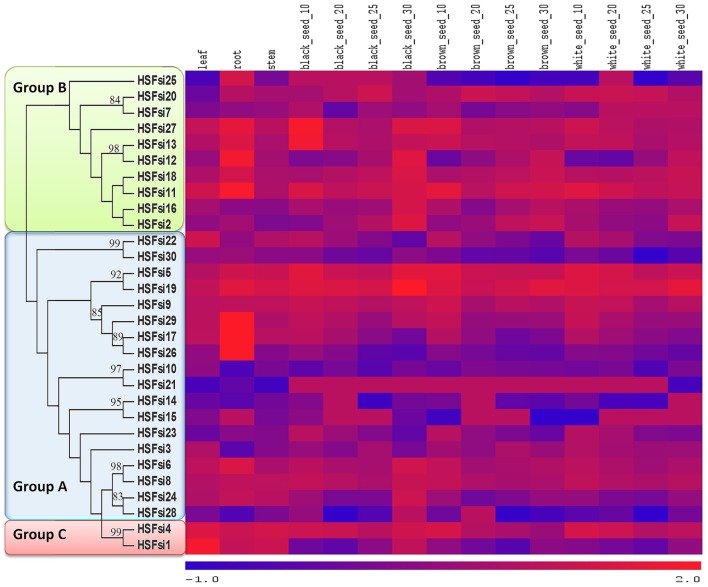
**Expression profile cluster analysis of the sesame Hsf genes in different organs**. Expression values of each Hsf gene were downloaded from RNA-seq data of 15 organs including roots, stems, leaves, and seed at different stage of development.

The expression patterns of sesame Hsf genes in different organs and tissues viz. root, leaf, stem tip, seed with different coat colors harvested at various development stages, were investigated. Heat maps using both phylogenetic tree and hierarchical clustering methods were generated based on the RPKM values for each gene in all samples (Figure 7, Supplementary Figure 1). The results showed that almost all sesame Hsf genes were expressed in the different tissues and organs. The hierarchical cluster analysis divided the 30 genes into five groups which were a mix of genes from different Hsf classes. Interestingly, expression levels differed from organ to organ and from Hsf gene to another even in the same class. For instance, *HSF4* were expressed highly in all tissues and organs while *HSF1* belonging to the same class as *HSF4* displayed a strong expression level in root, leaves and stem but low expression levels in seeds. The result suggests that just because the phylogenetic relationships of these genes are close doesn't mean they may develop similar biological functions. It is worth noting that most of genes displayed higher expression in root compared to the other tissues. This is understandable since the root is a fast-growing tissue whose metabolism and development processes are all more active than in other tissues. Moreover, Hsf genes displayed mostly their lowest expression levels in sesame seeds. Finally, the Hsf class B members appeared to be the most expressed genes in organs and tissues of sesame compared to the other two classes.

### Analysis of expression patterns of Hsf genes under different drought stresses

**Figure 8:**
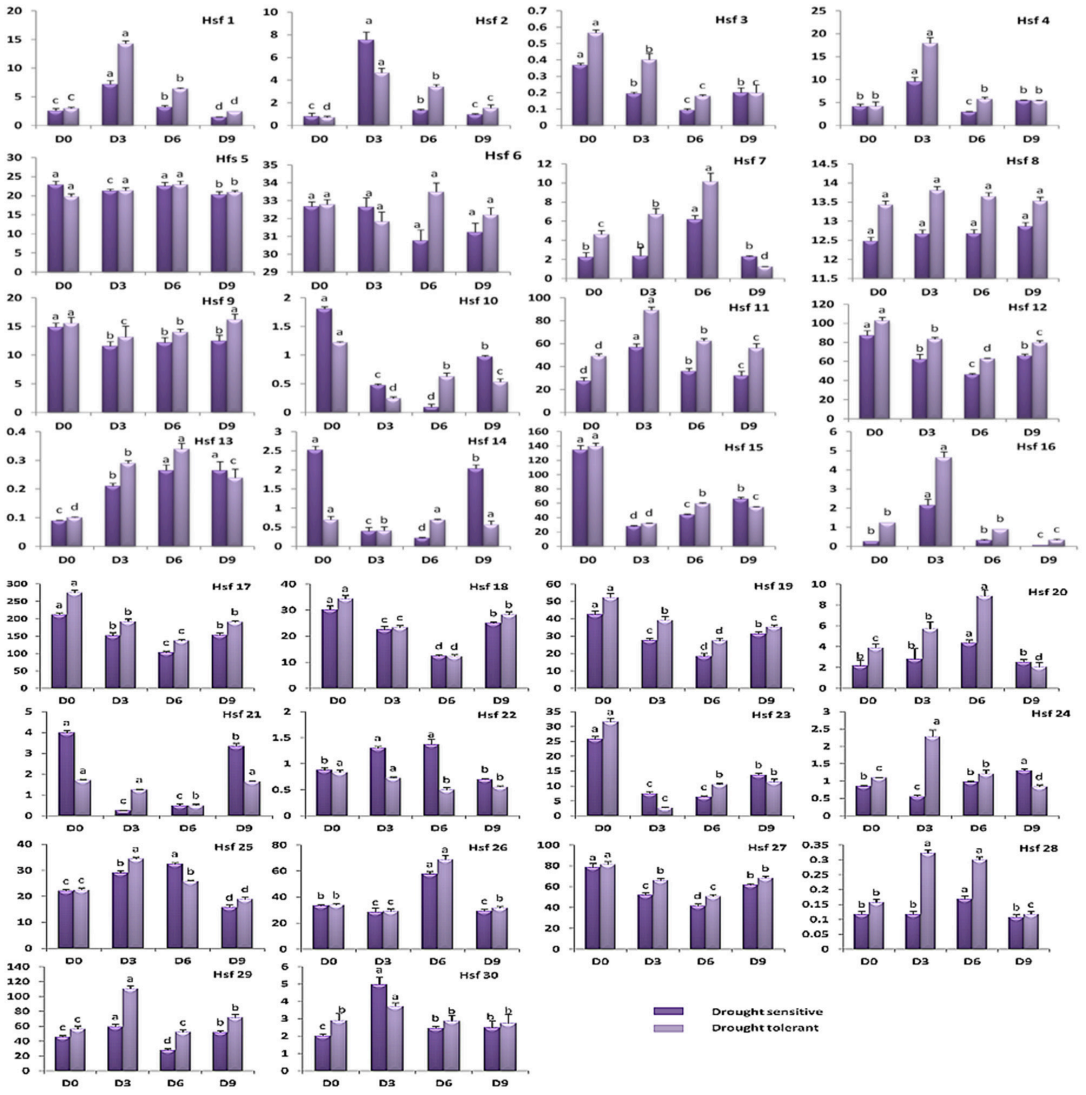
**Relative quantitative expression levels of sesame Hsf genes at a series of time points during drought stress treatments and after re-watering**. The experiments were repeated three times and gave consistent results. The mean values and SDs were obtained from three biological and three technical replicates (error bars indicate standard deviation). Bars of the same accession showing the same letter are not significantly different at 5% probability level.

To understand the potential role of Hsf genes in sesame responses to drought, we monitored all sesame Hsf genes expression levels during progressive drought and after re-watering in two contrasted accessions (drought-tolerant and drought-sensitive). Since most Hsf genes displayed higher expression in root compared to the other tissues, qRT-PCR analysis was performed in sesame root samples. The results showed that some genes were highly expressed while others had low expression levels and revealed 3 types of expression patterns: (1) Up-regulated genes, (2) down regulated genes, and (3) no regulated genes (Figure 8). In the first category (1), these genes showed increase of their expression levels during drought stress. This increase of expression levels could be observed early at the 3rd day (*HSF1, HSF2, HSF4, HSF11, HSF16, HSF22, HSF24, HSF25, HSF28, HSF29*, and *HSF30*), or tardily at the 6th day (*HSF7, HSF13, HSF20*, and *HSF26*). For the second category of genes (2), they displayed the decrease of their expression levels under drought stress (*HSF3, HSF10, HSF12, HSF14, HSF15, HSF17, HSF18, HSF19, HSF21, HSF23*, and *HSF27*). Hence, in most cases, while the expression levels of the genes in category (1) decreased after re-watering period, the genes in category (2) increased their expression levels. Finally, the genes in category (3), showed minor fluctuations during drought stress and after re-watering (*HSF5, HSF6*, and *HSF8*).

Comparing expression patterns of Hsf genes in the drought-tolerant accession and the drought-sensitive one, results showed that most of time, the expression levels of Hsf genes were broadly higher in the drought tolerant accession. Overall, it appeared that regulation by drought is not specific to some Hsf classes but depends on the genes.

## Discussion

### Relatively high number of Hsf genes in sesame genome resulted from recent segmental duplication

Benefiting from genome availability, Hsf gene family has been extensively studied in many crops. Twenty five genes were reported in *Arabidopsis* (Jin et al., 2014), 35 in Chinese cabbage (Song et al., 2014), 25 in rice (Guo et al., 2008), 26 in tomato (Yang et al., 2016), 22 in *U. gibba* (Jin et al., 2014), 27 in potato (Tang et al., 2016), 25 in maize (Lin et al., 2011), 29 in Chinese white pear (Qiao et al., 2015), 25 in Chinese pepper (Guo et al., 2015) etc., showing the multiplicity of this gene family in plants. In this study, we reported 30 Hsf genes in sesame genome, classified into 3 major classes. As reported in other crops, the class A genes are the most represented, followed by the class B members (Guo et al., 2008, 2015; Lin et al., 2011). However, no Hsf member belonging to the class A9 (At5g54070) was found in sesame similarly as reported in Chinese cabbage (Huang et al., 2015).

Compared to its immediate relatives *U. gibba* (~82 Mb), potato (~840 Mb), tomato (~950 Mb) and the model plant *Arabidopsis* (~135 Mb), sesame (~370 Mb) bears more Hsf genes in its genome, suggesting that the number of Hsf genes is not necessarily linked to genome size but might be explained by the different evolutionary histories occurred within each species. Whole genome duplications have had a strong impact on species diversification and may have triggered evolutionary novelties (Jaillon et al., 2009). WGDs within the angiosperms presumably are the cause of varying numbers of Hsfs between different plant species (Scharf et al., 2012). Sesame is estimated to have diverged from the tomato-potato lineage approximately 125 MYA (million years ago) and from *U. gibba* approximately 98 MYA (Wang et al., 2015). Tomato-potato lineage has recently experienced a whole genome triplication (WGT) and it was expected to have higher copies of Hsf genes in their genomes while sesame and *U. gibba* underwent independently a WGD at different points of evolution. Hence, we hypothesized that there might be some extensive Hsf genes loss after WGT events principally in the tomato-potato lineage. Conversely, known as arid areas crop, sesame might retain for adaptation purposes a large part of its Hsf genes during evolution. These observations are consistent with Lin et al. (2014) who demonstrated that the vast majority of Hsf gene duplications resulted from whole genome duplication events rather than tandem duplication and significant differences in gene retention exist from species to species.

To identify the evolutionary origins of Hsf genes in sesame, we further analyzed the distribution of the duplicated genes. Seven pairs of duplicated genes were found in sesame Hsf family, which was less than potato (8 pairs) but higher than tomato (4 pairs). Six gene pairs are involved in segmental duplication while the pair of genes *HSF6* and *HSF14* is involved in regional duplication within the LG6 and has probably evolved from their common ancient gene through the gene duplication event within this LG. These results suggest that segmental duplications were the primary force underlying the expansion of Hsf genes in sesame. Our finding corroborates well the previous reports in other plants about expansion of Hsf genes thought to result primary from segmental duplication (Lin et al., 2011, 2014; Song et al., 2014; Guo et al., 2015). Because most plants with diploidized polyploids retained numerous duplicated chromosomal blocks within their genomes, segmental duplication occurred more frequently than tandem duplication and transposition events in plants (Cannon et al., 2004). Based on synonymous substitution rates (Ks) which is a common procedure to determine the evolutionary age and divergence level of gene copies (Wang et al., 2013), these duplication events date back to around 67 MYA, similarly as predicted by Wang et al. (2015) who proposed a period between 54 and 72 MYA.

### Diversified roles of Hsf genes in sesame uncovered by gene orthology analysis and gene expression profiling

The close relationship between sesame and the well-studied plant *Arabidopsis* helped in identifying some orthologous genes and allowed us to predict their functions in sesame. Indeed, gene orthology analysis can be used as a preliminary method to explore the function of candidate genes (Wang M. et al., 2014). Only class A and class B genes were found to have orthologs Hsf in *Arabidopsis* genome and in-depth screening of these orthologs revealed two main groups of Hsfs: the most represented are those involved in regulation of transcription of others genes and few are involved in responses to heat and other abiotic stresses. Hanada et al. (2008) reported that transcription factors encoding nucleic acid binding proteins originated mostly through segmental duplication. Hence, we deduced that segmental duplication events in the expansion of Hsf genes in sesame may induce transcriptional regulation functions to these genes. For instance the gene *HSF2, HSF8, HSF9, HSF16*, and *HSF27* are orthologs of *Arabidopsis* genes (*AT2G26150, AT4G36990*, and *AT132330*) reported to act as activator or repressor of heat inducible Hsf genes in *Arabidopsis* (Ikeda et al., 2011; Weng et al., 2014). Furthermore, based on the interalog of *Arabidopsis*, the protein-protein interactions of some sesame Hsf genes were constructed, and results showed that most of genes interact with Hsps. Hsf genes have been demonstrated to play key roles in the tolerance to various stresses by reacting with different genes especially Hsps (Swindell et al., 2007; Hu et al., 2009). Unfortunately, the regulation of the Hsps by the Hsf genes in sesame is still not documented and future works should focus on this issue to unravel the mechanisms of regulation as well as the possible interaction with other transcription factors such as DREB2A as demonstrated by Scharf et al. (2012).

Some authors described Hsf genes as tissue- and stage-specific in several plants (Swindell et al., 2007; Giorno et al., 2010). This was not the case in our study and, as previously reported in *L. japonicus* (Lin et al., 2014), all sesame Hsf genes were expressed in all organs and tissues investigated, suggesting the functional conservation of this gene family and their regulatory roles at multiple developmental stages. However, they displayed different expression levels, pointing out their functional differences. Data also showed that some duplicated genes exhibited different expression patterns in organs and tissues of sesame. This is, for instance, the case of the duplicated genes *HSF1* and *HSF4*, indicating that as a major feature of the long-term evolution, duplicated genes have diversified their functions (Blanc and Wolfe, 2004). Class B-Hsfs and class A-Hsfs genes were the most abundant in the different tissues and organs in sesame, showing their active roles in vegetative growth as well as seed maturation. Similar expression patterns of these genes were reported in barley, potato and legumes (Lin et al., 2014; Reddy et al., 2014; Tang et al., 2016).

Sesame is primarily grown for its seeds which bear one of the highest oil content. Thus, genes involved in sesame seed maturation are of great importance. Surprisingly, we detected that sesame genome lacks HsfA9 group which has been described to play a specific role in seed development in many species like *Arabidopsis*, tomato and tobacco (Kotak et al., 2007; von Koskull-Döring et al., 2007). Such observation implies that sesame might hold other functional genes which perform this parallel function. For instance, *HSF4, HSF9, HSF11*, and *HSF15* exhibited abundant transcripts in sesame seeds whichever the seed color or the development stage. Hence these genes are likely to play key roles in sesame seed development. In rice, OsHsfA7 has also been proposed to play the seed-specific function (Chauhan et al., 2011).

### Hsf genes are actively involved in drought response in sesame

The major objective for agronomic research is to enhance crop productivity under various abiotic stresses (Puranik et al., 2012). Heat stress is often compounded by additional abiotic stresses such as drought and salt stress (Bita and Gerats, 2013). Although it is well known that Hsf genes are involved in the acclimation of plant to heat, other stresses such as drought are less documented.

In this study, two contrasting accessions of sesame based on their response to drought were used for time-course Hsf genes expression and 90% of these genes were drought responsive. This result indicated the involvement of Hsfs in drought stress tolerance in sesame, which is in agreement with previous studies in different plant species (Chauhan et al., 2011; Bechtold et al., 2013; Li et al., 2014). In addition, we observed that most of these drought responsive Hsf genes in this study are highly expressed in the drought-tolerant accession compared to the sensitive one, confirming their key roles in drought tolerance. About 50% of Hsf genes were up-regulated while 36% were down-regulated, showing that these genes might play specific roles and sesame adapts to drought stress through compensation and interaction of its Hsf genes expression. Most of down-regulated genes were class A-Hsfs, while many up-regulated genes belonged to the class B and C. This result is intriguing, knowing the importance of some class A-Hsfs such as HsfA2 and HsfA1 members which have been identified to be responsive to heat stress in *Arabidopsis* and tomato (Mishra et al., 2002; Ogawa et al., 2007) and may probably be involved in other abiotic stresses such as drought (Ogawa et al., 2007; Bechtold et al., 2013; Zhang et al., 2015). However, under drought stress, most of these genes were down-regulated in sesame. In the works of Guo et al. (2015), they also highlighted the involvement of some class B-Hsfs in osmotic stress tolerance in pepper. More notably, they observed that class A-Hsfs expressed differentially under various stresses. In soybean, the Hsf type-A1 gene GmHsf-17, was strongly induced under heat stress but not influenced under drought (Li et al., 2014). These results indicate that there are species-specific and stress-specific features in the functions of Hsf members in regulating genes involved in plant stress responses and we propose class B-Hsfs as major transcriptional repressors or co-activators cooperating with some class A-Hsfs (Bharti et al., 2004; Scharf et al., 2012), to confer drought resistance in sesame.

Although HSF genes are well characterized in the heat stress-related pathway, they are poorly understood in other stress responses in *Arabidopsis* (Huang et al., 2016). For instance, the few studies focused on drought stress revealed the role of HsfA6b, HsfA6a, and HsfA1b in drought stress responses in *Arabidopsis* (Bechtold et al., 2013; Hwang et al., 2014; Huang et al., 2016). Through syntheny analysis between sesame and *Arabidopsis*, we found the gene *HSF10* as orthologs of HsfA6b in *Arabidopsis, HSF19* as orthologs of HsfA1b in *Arabidopsis* while no orthology was found for the gene HSFA6a in sesame. Gene expression analysis of these 2 genes (*HSF10* and *HSF19*) in sesame revealed that they were significantly down regulated under progressive drought stress in the resistant accession as well as in the sensitive one. This suggests that these genes might also be involved in drought response pathways in sesame.

To cope with prolonged drought stress, sesame uses different set of Hsf genes belonging to class B4, class B3 and class A1 to maintain housekeeping gene expression. In *Arabidopsis*, similar pattern has been reported under heat stress with AtHsfA1a and AtHsfA1b involved in the early response to heat stress (Lohmann et al., 2004) while AtHsfA2 enhanced and maintained the response when plants are subjected to long-term stress (Charng et al., 2007). The two members of Class C-Hsfs were induced under drought stress in sesame and may play important function during drought stress. In contrast to class A-Hsfs and class B-Hsfs, members of class C have not so far been fully characterized. Further functional works are needed to excavate the role of these genes in drought tolerance in sesame. The unaltered genes (all belonging to the class A-Hsfs) might operate on the regulation pathways of other abiotic stresses (Victor and Benecke, 1998). During plant recovery from drought damage, the up-regulated genes expression levels slowly decreased while the down-regulated genes increased. These results imply that the functions of down-regulated genes are more potent under normal conditions in sesame plants. However, gene expression is a complex biological process and more thorough studies are needed to decipher the regulatory mechanisms between Hsfs and their co-expressed genes not only under drought stress but in combination with other stresses.

## Conclusion

The importance of Hsf gene family has attracted many investigations on different crops. This study presents a comprehensive overview of this gene family in sesame. Here, we reported 30 non-redundant full length Hsf genes in the sesame genome. Structural characteristics and the comparison with *Arabidopsis* and *U. gibba* helped to classify these genes into 3 major classes with members of class A the most abundant. These genes were unevenly distributed in 12 of the 16 LGs. Segmental duplication events contributed to the expansion of Hsf genes and have played key roles in their functional divergence. All sesame Hsf genes were found expressed differentially in all tissues and organs examined according to the RNAseq data analysis.

Sesame is the model plant for oil crops with small genome size and high oil content in its seed. Drought is one of the major constraints which impair its production. Therefore, understanding the role of stress related genes such as Hsf in sesame is of a significant meaning.

Expression analysis of Hsf under drought stress showed differences in their expression patterns, confirming this gene family as potential candidate for improvement of drought tolerance in sesame. The class B-Hsfs were the most expressed ones under drought stress and we propose these genes for future gene cloning and functional analyses toward the improvement of drought and other abiotic stresses tolerance in sesame cultivars.

## Author contributions

KD carried out the bioinformatics, experiments, data analysis and drafted the manuscript. DD participated in data analysis and revised the manuscript. NC supervised the experiments and revised the manuscript. All authors have read and approved the final manuscript.

### Conflict of interest statement

The authors declare that the research was conducted in the absence of any commercial or financial relationships that could be construed as a potential conflict of interest.

## Supplementary material

Supplementary Table 1 **List of primers used in quantitative real time-PCR expression analysis**.

Supplementary Table 2 **Characteristics of sesame Hsf Transcription factor gene family members**. This table contains the type, distribution of Hsf domains, protein full length, subfamily, ORF length, number of CDS, lists of predicted domains.

Supplementary Table 3 **The Ka/Ks ratios and estimated duplication time for duplicated sesame Hsf genes**.

Supplementary Table 4 **Motif sequences identified using MEME tools in sesame Hsf genes**.

Supplementary Table 5 **Orthologous gene pairs of Hsfs and their localization in sesame and ***Arabidopsis*** genomes**.

Supplementary Table 6 **Details of sesame AP2/ERF transcription factor-based SSR markers**.

Supplementary Figure S1 **Hierarchical clustering heat map of the expression of Hsf genes in sesame organs**. Expression values of each Hsf gene were downloaded from RNA-seq data of 15 organs including roots, stems, leaves, and seed at different stage of development.
