# Supplementary figures and images for: Genome-Wide Investigation of Hsf Genes in Sesame Reveals Their Segmental Duplication Expansion and Their Active Role in Drought Stress Response
Source: Front Plant Sci. 2016 Oct 13;7:1522. doi: 10.3389/fpls.2016.01522 (PMC5061811; doi:10.3389/fpls.2016.01522)

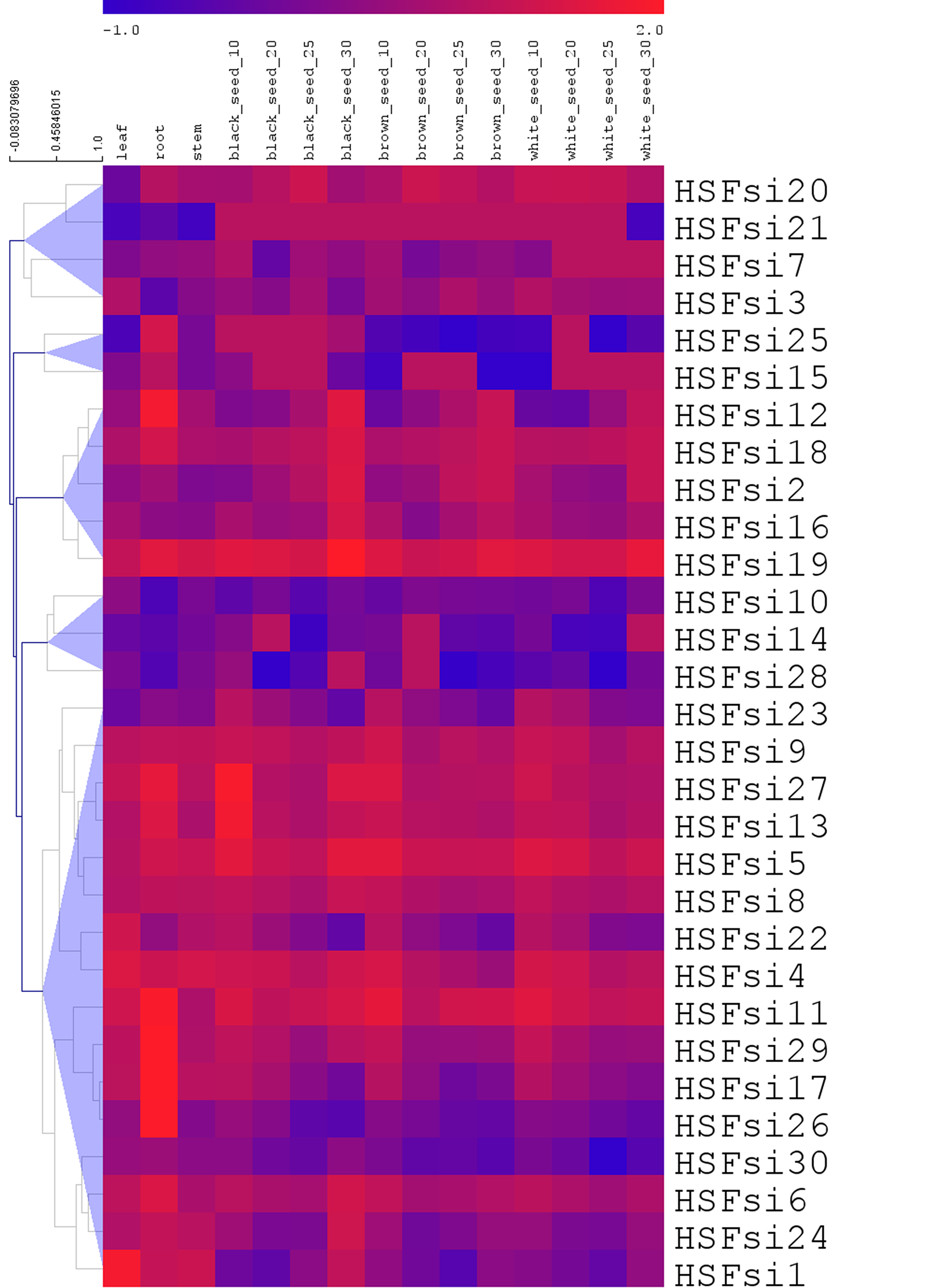

Supplement: Supplementary Figure S1 — Hierarchical clustering heat map of the expression of Hsf genes in sesame organs. Expression values of each Hsf gene were downloaded from RNA-seq data of 15 organs including roots, stems, leaves, and seed at different stage of development. [file Image1.TIF]
